# Effects of *Rhododendron* removal and prescribed fire on bees and plants in the southern Appalachians

**DOI:** 10.1002/ece3.8677

**Published:** 2022-03-01

**Authors:** Michael Ulyshen, Katherine Elliott, Joel Scott, Scott Horn, Patsy Clinton, Ning Liu, Chelcy F. Miniat, Peter Caldwell, Chris Oishi, Jennifer Knoepp, Paul Bolstad

**Affiliations:** ^1^ 124330 USDA Forest Service Southern Research Station Athens Georgia USA; ^2^ USDA Forest Service Coweeta Hydrologic Laboratory Otto North Carolina USA; ^3^ USDA Forest Service Eastern Forest Environmental Threat Assessment Center Research Triangle Park North Carolina USA; ^4^ USDA Forest Service Rocky Mountain Research Station Albuquerque New Mexico USA; ^5^ Department of Forest Resources University of Minnesota St. Paul Minnesota USA

**Keywords:** Apoidea, conservation, forest management, pollinators, restoration

## Abstract

*Rhododendron maximum* is an evergreen shrub native to the Appalachian Mountains of North America that has expanded in recent decades due to past disturbances and land management. The purpose of this study was to explore how bees and plants were affected by the experimental removal of *R*. *maximum* followed by a prescribed fire in one watershed compared to a neighboring reference watershed. Bees and plants were sampled for three years in both watersheds. Comparisons were based on the rarefaction and extrapolation sampling curves of Hill numbers as well as multivariate methods to assess effects on community composition. Bee richness, Shannon's diversity, and Simpson's diversity did not differ between watersheds in the year after removal but were all significantly higher in the removal watershed in year two, following the prescribed fire. Bee Shannon's diversity and Simpson's diversity, but not richness, remained significantly higher in the removal watershed in the third year. Similar but weaker patterns were observed for plants. Comparisons of community composition found significant differences for bees in the second and third year and significant differences for plants in all three years. For both groups, significant indicator taxa were mostly associated with the removal watershed. Because bees appeared to respond more strongly to the prescribed fire than to the removal of *R*. *maximum* and these benefits weakened considerably one year after the fire, clearing *R*. *maximum* does not appear to dramatically improve pollinator habitat in the southern Appalachians. This conclusion is underscored by the fact that about one quarter of the bee species in our study area were observed visiting *R*. *maximum* flowers. The creation of open areas with wildflowers may be a better way to benefit bees in this region judging from the high diversity of bees captured in the small roadside clearings in this study.

## Graphical abstract

We used a paired watershed approach to investigate the effects of *Rhododendron maximum* removal and prescribed fire on bees and plants in the southern Appalachians. The treatments increased the diversity of both groups but these effects weakened one year after the fire. Based on bees collected from flowers, we estimate that a quarter of the bee species in our study area utilize *R. maximum* blooms.

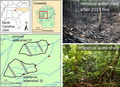

## 1 INTRODUCTION

Native bees and other pollinators within the historically forested regions of North America have experienced considerable changes since European colonization. Among these, the near‐complete conversion of old growth forests to agriculture, development, or second‐growth stands was particularly transformative (MacCleery, 1993). Studies exploring the relationship between surrounding forest cover and bee diversity have yielded mixed results. While some report that forest cover has a positive effect on the diversity of bees (Bennett & Isaacs, 2014; Taki et al., 2007; Watson et al., 2011), others suggest the opposite (Winfree et al., 2007). Such discrepancies may be driven by differences in habitat associations among bee taxa. For example, Smith et al. (2021) found that about a third of all bees in the northeastern United States are associated with forests. Another third were open‐habitat species while the remainder were classified as habitat generalists. Although previous studies from that region have shown a negative relationship between forest cover and total bee diversity (Winfree et al., 2007), Smith et al. (2021) reported a positive relationship between the amount of forest area and the diversity of forest‐associated species. Moreover, Harrison et al. (2018) concluded that forest bees are largely replaced in agricultural and urban environments by species adapted to open habitats. Such findings suggest that forests play a key role in conserving bee diversity and underscore the importance of research aimed at optimizing forest conditions for these important and imperiled insects.

Today, forests in the United States cover about two thirds of the area that was forested in the year 1600 but differ from those earlier forests in many respects (MacCleery, 1993). The introduction of non‐native species, fire suppression, fragmentation, and altered stand density have all changed forest composition and structure. It is apparent from a growing body of literature that native bees are sensitive to forest conditions (Hanula et al., 2016). One of the clearest patterns is that open stand conditions promote more diverse bee communities, in part through increasing floral resource availability near the forest floor (Hanula et al., 2016). As a consequence, more open early successional forests often support higher bee diversity than older forests with more closed canopies (Rivers & Betts, 2021; Taki et al., 2013). In mature forests, a number of management interventions have been shown to result in more open conditions and higher bee diversity. These include stand thinning, prescribed fire, and the mechanical removal of invasive shrubs (Hanula et al., 2016; Ulyshen et al., 2021). Invasive shrubs can include non‐native species that dominate the midstory and largely exclude herbaceous plants from the forest floor as well as native species that have increased in abundance due to past disturbances and management history. One example of the latter concerns *Rhododendron maximum* L., an evergreen ericaceous shrub native to the Appalachian Mountains of the eastern United States. This shrub now dominates the forest midstory over large areas due to a combination of logging, the loss of American chestnut (*Castanea dentata* (Marsh.) Borkh.) and Eastern hemlock (*Tsuga canadensis* (L.) Carr.) from the overstory, and fire suppression (Elliott & Vose, 2012; Ford et al., 2012; Van Lear & Brose, 2002). Herein, we present the results from a study aimed at assessing the effects of *R*. *maximum* removal followed by prescribed fire on bee communities in North Carolina.

The Southern Appalachian region is considered a biodiversity hotspot, supporting North America's highest diversity of trees as well as many other plant and animal species (Stein et al., 2000). However, disturbances have altered forest structure and species composition of southern Appalachian watersheds (Adams, 2013; Bearup et al., 2014; Brantley et al., 2013, 2015; Ice et al., 2004). These include pre‐ and early settlement fires, logging in the early 20th century, drought, hurricanes, and insect and disease outbreaks. Most notable among these outbreaks was the introduced chestnut blight fungus (*Endothia parasitica* (Murr.) P.J. And. & H.W.) in the 1920s–1930s that led to widespread mortality of American chestnut. Consequently, overstory tree species composition changed from dominance by American chestnut in the 1930s, to drought‐tolerant, xerophytic oaks (*Quercus* spp.) by mid‐century, and finally to dominance by drought‐intolerant, mesophytic species such as red maple (*Acer rubrum* L.) and tulip poplar (*Liriodendron tulipifera* L.) by the end of the century (Elliott & Swank, 2008; Elliott & Vose, 2011; Ford et al., 2012; Nelson, 1955). Subcanopy species composition changed along with the canopy changes, including the expansion of *R*. *maximum*. *Rhododendron maximum* is an important species in these forests as it is highly shade tolerant, forms a dense subcanopy that shades the forest floor (Clinton, 2003), has little to no herbaceous or tree seedling (henceforth, understory) cover below its subcanopy (Beckage et al., 2000; Clinton et al., 1994), and it decreases N availability in the soil and litter layer to nonericaceous species (Wurzburger & Hendrick, 2007, 2009). As a result, tree height, biomass, and productivity are substantially reduced in areas with dense *R*. *maximum* cover (Bolstad et al., 2018). With regard to foraging bees and other pollinators, *R*. *maximum* flowers prolifically in the early summer months. Although the flower‐visiting insect communities associated with other species of *Rhododendron* have been documented in previous studies from Europe and Asia (Sugiura, 2012; Tiedeken & Stout, 2015), no previous effort, to our knowledge, has been made to characterize the bee community visiting *R*. *maximum* in North America. This is a particularly interesting question given that the nectar produced by some *Rhododendron* species contains secondary compounds known to be toxic to some bee species (Tiedeken et al., 2016).

In this study, we used a paired‐watershed study design to investigate the effects of *R*. *maximum* removal followed by prescribed fire on the diversity of bees and herbaceous plants. Based on the literature summarized above, we hypothesized that the diversity of both groups would be significantly higher in the removal watershed and that the strongest effects would be detected after the prescribed fire. We also predicted that individual bee species would show positive associations with the removal watershed but not the reference watershed. We also sought to better understand the value of *R*. *maximum* to the local bee community by sampling bees directly from the flowers of this shrub.

## 2 METHODS

### 2.1 Study area and treatment

**FIGURE 1:**
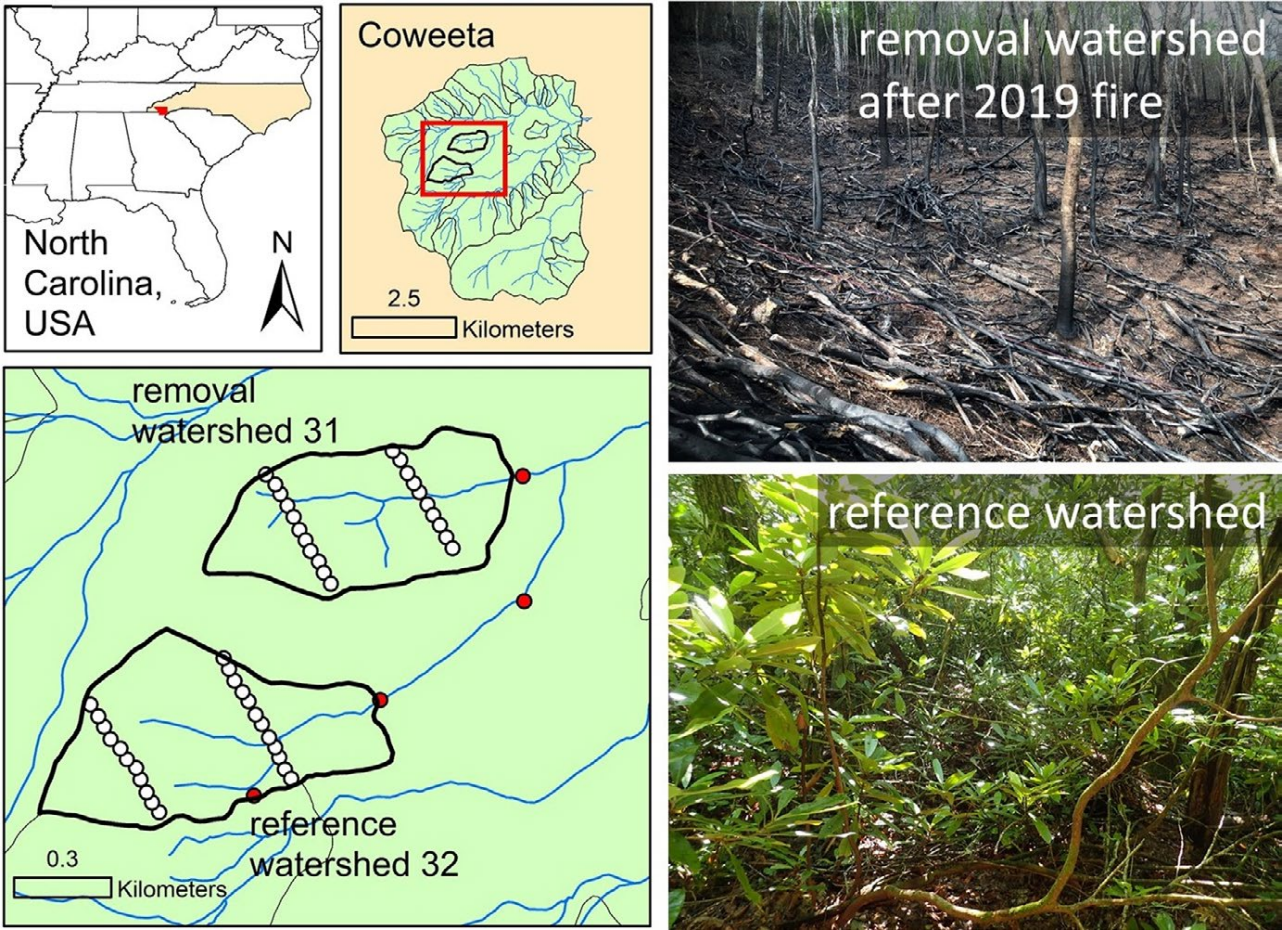
Map showing locations of the experimental watersheds used in this study. White dots represent locations where bees and plants were sampled within the watersheds. Red dots represent roadside forest openings where bees were sampled in 2019. Photos on the right show conditions in the removal watershed soon after the 2019 fire (top) and a *R*. *maximum* thicket in the reference watershed (bottom)

This study was conducted at the Coweeta Hydrologic Laboratory (henceforth Coweeta) in the southern Appalachian Mountains of western North Carolina (Figure 1). Established in 1934, Coweeta is part of the USDA Forest Service Experimental Forest and Ranges network and was also a Long‐term Ecological Research (LTER) network site. Oak–hickory forests dominate the lower elevations while higher elevations are characterized by northern hardwoods, both with increasing dominance of mesophytic species (e.g., red maple, tulip poplar) in the overstory and *R*. *maximum* in the subcanopy (Elliott & Swank, 2008; Elliott & Vose, 2011).

We utilized an ongoing study aimed at cutting *R*. *maximum* followed by prescribed fire as a way to increase water yield and understory plant diversity. The design follows the paired‐watershed approach (Wilm, 1943) involving two neighboring watersheds with similar climate, vegetation, stand age, aspect, elevation, topography, and soils. The strength of this design is that the treatment effect can be isolated by accounting for year‐to‐year variation in climate using the reference watershed. The “removal watershed” is east‐facing and mid‐elevational (869–1141 m) with an area of 37.8 ha while the “reference watershed” is also east‐facing and mid‐elevational (920–1236 m) and 41.3 ha in area. Although neither watershed was uniformly covered by *R*. *maximum* at the beginning of the study, there was no significant difference in cover between the two watersheds initially based on observations at our 44 sampling plots (*χ* ^2^ = 0.76, *p* = .38). Another shrub, mountain laurel (*Kalmia latifolia* L.) was also present on the ridges in both watersheds and was not cut except in cases where it overlapped with *R*. *maximum*. As the Coweeta basin was last logged in the 1920s, before Coweeta Hydrologic Lab was established in 1934, both watersheds had been largely undisturbed for about a century or more prior to this study.

*Rhododendron maximum* stems were felled with chainsaws in the removal watershed beginning in September of 2017 and completed by January of 2018 (Table 1, Figure 1). Cut limbs were left on site and piled <1.2 m high. Stumps were sprayed with a solution of 50% triclopyr amine (Garlon 3A^®^, DOW AgroSciences, Indianapolis, IN) with an aquatic label to avoid resprouting (Elliott & Miniat, 2018). After the cut stems and slash had more than a year to dry, the same watershed was subjected to a prescribed fire on 23 March 2019 (Table 1). The reference watershed was not manipulated and served as the experimental control.

**TABLE 1:** Timeline of activities 2017–2020

	2017	2018	2019	2020
*R*. *maximum* removed from removal watershed	September–December	January		
Prescribed fire in removal watershed			March	
Bee sampling, both watersheds		May, June, August	April, May, June	April, May, June
Plant sampling, both watersheds		July	June	June
Bee sampling, *R*. *maximum* flowers		June	June	June
Bee sampling, open roadside clearings			April, May, June	

### 2.2 Bee and plant sampling

We sampled bees and recorded herbaceous plant diversity in long‐term permanent vegetation plots that were established in the 1930s. The plots within each watershed were arranged in two transects separated by ~400 m, and ~40 m separated our sampling locations within each transect. There were 23 and 21 plots in the reference and removal watersheds, respectively. To sample bees, we put out a set of three colored pan traps (white, yellow, and blue) in each plot, separated by 1 m and placed in a line parallel to the transect (Appendix 1). Although pan traps are known to sample some taxa more effectively than others (O'Connor et al., 2019), they provide a highly standardized method for sampling pollinators in forests. The traps were held ~20–30 cm from the ground on wire stands and were filled with soapy water when in operation. For each sampling period, traps were collected after three days, pooled by plot, and returned to the lab for identification. We sampled in May, June, and August in 2018 and found bee numbers to drop quickly as the season progressed, a trend common for bees in temperate deciduous forests (Harrison et al., 2018). Therefore, we sampled monthly from April to June in 2019 and 2020 (Table 1). All plants growing <0.5 m in height (regardless of flowering status) were recorded within a permanent 1 m^2^ quadrat situated near the pan traps in late June to early July of each year (Table 1). Although the number of individuals and/or percent cover was counted or estimated for each observed plant species, only presence/absence data were used in this study.

To better understand the value of open areas to bee communities in our study area, we used the same methods to sample bees in four roadside clearings in 2019 (Figure 1, Appendix 1). To determine the importance of *R*. *maximum* as a floral resource, we sampled bees visiting *R*. *maximum* flowers using nets. This involved spending 2–4 person‐hours per year sampling *R*. *maximum* blooms at various roadside locations within or adjacent to the experimental watersheds. Although *R*. *maximum* does bloom under full canopy cover (Appendix 2), we focused on roadside locations due to greater availability and ease of access. We acknowledge that bee visitation of *R*. *maximum* flowers in open areas may not accurately reflect visitation within the forest but feel this information can provide valuable insights into the importance of this shrub to the local bee fauna.

### 2.3 Plot measurements

To better understand how local conditions influence bee and plant diversity, we collected data on canopy openness and burn severity at each plot. To measure canopy openness, we first used fisheye lenses to photograph overhead canopy cover. In 2018, these images were taken with a camera mounted on a tripod at ~1 m above the ground. Because the mountainous terrain made this approach cumbersome, we used a fisheye lens attachment on a cellphone (~1.7 m above the ground) to capture images in subsequent years. Thus, canopy openness values from 2018 cannot be meaningfully compared to those taken in 2019 or 2020. We then used software (WinSCANOPY, regentsinstruments.com) to calculate canopy openness from each of these images. To obtain information on fire severity, we calculated the relative differenced normalized burn ratio (RdNBR, unitless) (Miller & Thode, 2007) at each of our plots using the Sentinel‐2 clear‐sky images (10 m × 10 m) before (18 March 2019) and five days after the prescribed fire (28 March 2019):

$$\text{RdNBR}\;=\frac{\left(\text{Prefire NBR}\;‐\;\text{Postfire NBR}\right)*1000}{\left(\sqrt{\left|\text{Prefire NBR}\right|}\right)}$$

The images used in this analysis were downloaded from Google Earth Engine Sentinel‐2 MSI: MultiSpectral Instrument, Level‐2A product (https://developers.google.com/earth‐engine/datasets/catalog/COPERNICUS_S2_SR). These images were atmospherically corrected to bottom of atmosphere surface reflectance using the algorithm “sen2cor.” The values were averaged using a 10‐m radius buffer centered on each plot. Values <70 were classified as unburned while those >600 are considered highly burned (Caldwell et al., 2020; Miller & Thode, 2007).

### 2.4 Rhododendron maximum cover

Because neither watershed was uniformly covered in *R*. *maximum*, we recorded which of the plots in the removal watershed had been covered by the shrub pre‐treatment based on the presence of cut *R*. *maximum* stems in the immediate vicinity of the bee sampling locations. For the reference watershed, we considered a plot to be partially covered by *R*. *maximum* if there was at least one stem >2.5 cm in diameter within a two‐meter radius of any of our bowl locations or any *R*. *maximum* leaves or branches visible directly above any of the pan traps. We then assigned each plot to one of three *R*. *maximum* cover categories: removed (*n* = 10); absent (*n* = 19); and present (*n* = 15).

### 2.5 Analysis

We first tested the abundance, richness, and Shannon's diversity of bees as well as plant richness and canopy openness for spatial autocorrelation. This was done by calculating Moran's I using the package ape (Paradis & Schliep, 2018) in R 3.6.1 (R Core Team, 2019). Because the variables were significantly autocorrelated (results not shown), and were thus not independent (an assumption of linear models), we used the iNEXT package (Hsieh et al., 2016) to compare bee and plant diversity estimates between the two watersheds based on the rarefaction and extrapolation sampling curves of Hill numbers. Hill numbers are a mathematically unified family of diversity indices (Chao et al., 2014) where the value of *q* determines how much weight is given to species based on their abundance. While *q* = 0 (richness) gives rare and abundant species equal weight, *q* = 1 (Shannon's diversity) gives more weight to abundant species and *q* = 2 (Simpson's diversity) counts only the dominant species (Hsieh et al., 2016). For both bees and plants, we analyzed sample‐based (incidence) data and compared diversity at the base sample size, which in this case was twice the minimal reference sample size (Chao et al., 2014). Differences are considered significant when 95% confidence intervals do not overlap at the base sample size. For both bees and plants, we repeated this analysis for the different *R*. *maximum* cover categories (i.e., removed, absent, and present) as described above.

Next, to better understand the relationship between canopy openness and the richness of bees and plants, we calculated Pearson's correlations with t‐tests to assess significance for each year separately. Similar comparisons were made between bee richness and plant richness as well as for burn severity and bee richness. We limited this last comparison to data from the removal watershed only as the reference watershed was not burned. Finally, we used PC‐ORD v.6 (McCune & Mefford, 2011) to investigate how bee community composition differed between watersheds for each year separately. To reduce the influence of highly abundant species, we relativized by species maxima (so that the abundance of each species ranged from 0 to 1) prior to analysis. We first performed nonmetric multidimensional scaling (NMDS) using the Bray–Curtis distance measure to produce ordinations for each year. We then conducted the multiple response permutation procedure (MRPP) to test for significant differences in community composition between treatments followed by indicator species analysis to determine which species were significantly associated with either treatment (Dufrêne & Legendre, 1997). Indicator values range from 0 (no indication) to 100 (perfect indication) and statistical significance is based on a Monte Carlo randomization test without corrections for multiple testing. The same analyses were performed on plants based on presence/absence data (and the Jaccard distance measure, where applicable). Although no useful NMDS ordinations were found for plants, MRPP and indicator species analysis were performed for each year separately.

## 3 RESULTS

### 3.1 Bees

**FIGURE 2:**
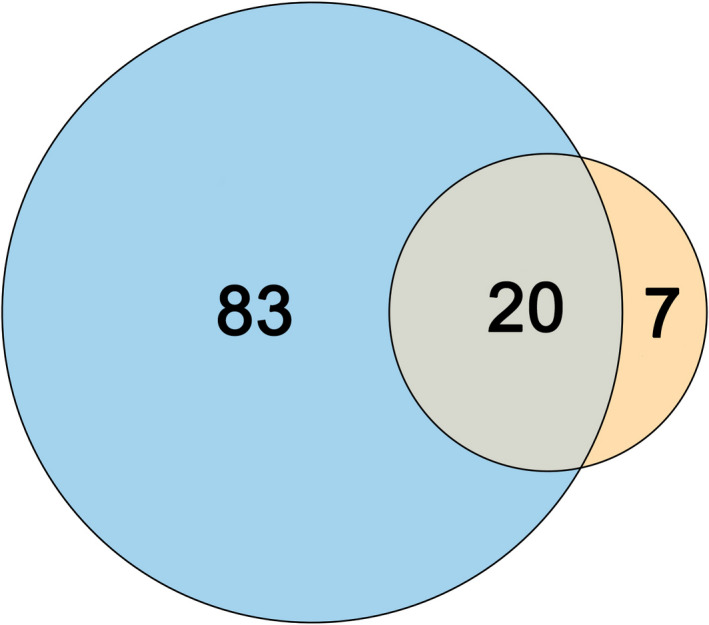
Venn diagram showing the number of species captured in both watersheds and neighboring open areas using pan traps (large circle), and from *R*. *maximum* flowers (small circle) or by both methods (area of overlap)

In total, 110 species of bees were captured in this study (3, 4 and 3, 4) of which a quarter were collected from *R*. *maximum* flowers (Figure 2, Appendix 4). The Chao1 richness estimate for the entire assemblage within our study area was 152.65 species (95% CIs: 126.75–218.59), about 24% of which were estimated to visit *R*. *maximum* flowers (37.01, 95% CIs: 29.34–69.73). Sampling at four roadside clearings in 2019 yielded 512 bee specimens from 66 species (Appendix 3).

**FIGURE 3:**
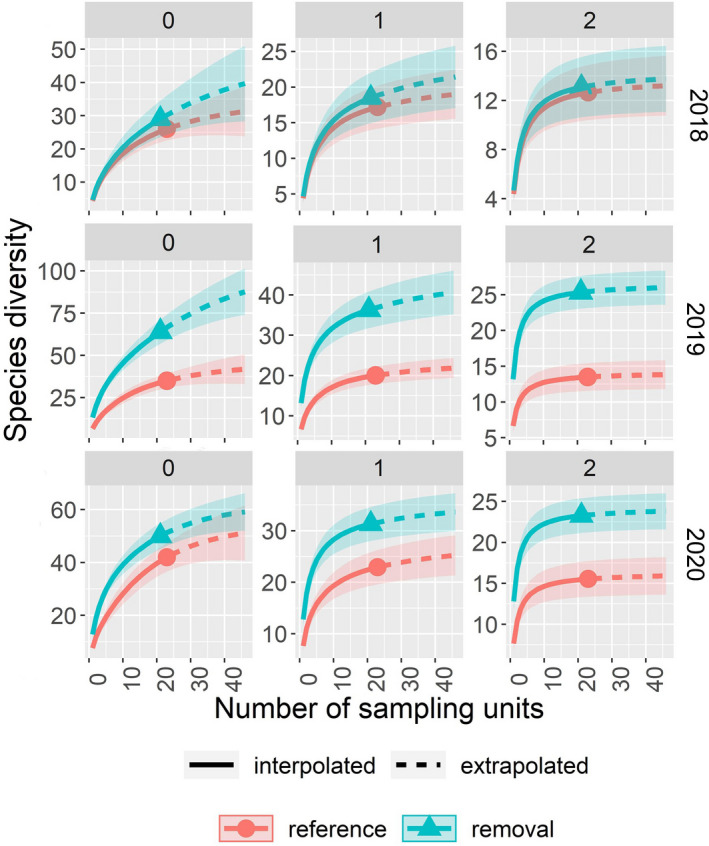
Rarefaction (solid lines) and extrapolation (dashed lines) of bee diversity in the reference and removal watersheds. Separate results are given for Hill numbers 0, 1, and 2. The results for species richness (*q* = 0) are shown in the left‐most panels, whereas those for Shannon's diversity (*q* = 1) and Simpson's diversity (*q* = 2) are shown to the right. All curves include 95% confidence intervals, and comparisons are made at 42 sampling units, that is, twice the smallest reference sample size

We found no differences in bee richness (*q* = 0), Shannon's diversity (*q* = 1), or Simpson's diversity (*q* = 2) in 2018, post‐cutting but prior to the prescribed burn, based on overlapping confidence intervals at the base sample size (Figure 3). However, all three metrics were significantly higher in the removal watershed following the prescribed burn in 2019. These patterns were repeated in 2020 except for *q* = 0, which did not differ significantly between treatments (Figure 3). Our separate comparison of *R*. *maximum* cover categories provides insights into the local effects of *R*. *maximum* cover on bees. There were no differences among the three categories in 2018 but plots from which *R*. *maximum* was absent or removed had significantly higher bee diversity at all levels of *q* than plots in which *R*. *maximum* was present in 2019 (Appendix 5). Except for no significant differences in species richness (*q* = 0), the results were similar in 2020. There was a tendency toward higher Simpson's diversity in the removed plots than in the absent plots, and this difference was significant in 2020. Bee richness increased with increasing plant richness in all three years (Table 2). In addition, bee richness increased with increasing canopy openness in 2019 and 2020 but not in 2018 (Table 2). Bee richness also increased with increasing burn ratio (RdNBR) in all three years in the removal watershed (Table 2).

**TABLE 2:** Pearson correlation coefficients and t‐test results for comparisons of bee richness, plant richness, canopy openness, and relative differenced normalized burn ratio (RdNBR) by year

	2018	2019	2020
Bee richness × plant richness	*r* = 0.34, *t*_42_ = 2.35*	*r* = 0.41, *t*_36_ = 2.71*	*r* = 0.43, *t*_42_ = 3.05**
Bee richness × canopy openness	*r* = −0.07, *t*_42_ = −0.42	*r* = 0.41, *t*_42_ = 2.92**	*r* = 0.54, *t*_42_= 4.16***
Bee richness × RdNBR	*r* = 0.45, *t*_19_ = 2.19*	*r* = 0.49, *t*_19_ = 2.48*	*r* = 0.45, *t*_19_ = 2.18*
Plant richness × canopy openness	*r* = −0.16, *t*_42_ = −1.03	*r* = −0.03, *t*_36_ = −0.15	*r* = 0.11, *t*_42_ = 0.70

Note

Asterisks denote significance: *p < .05; **p < .01; ***p < .001.

**FIGURE 4:**
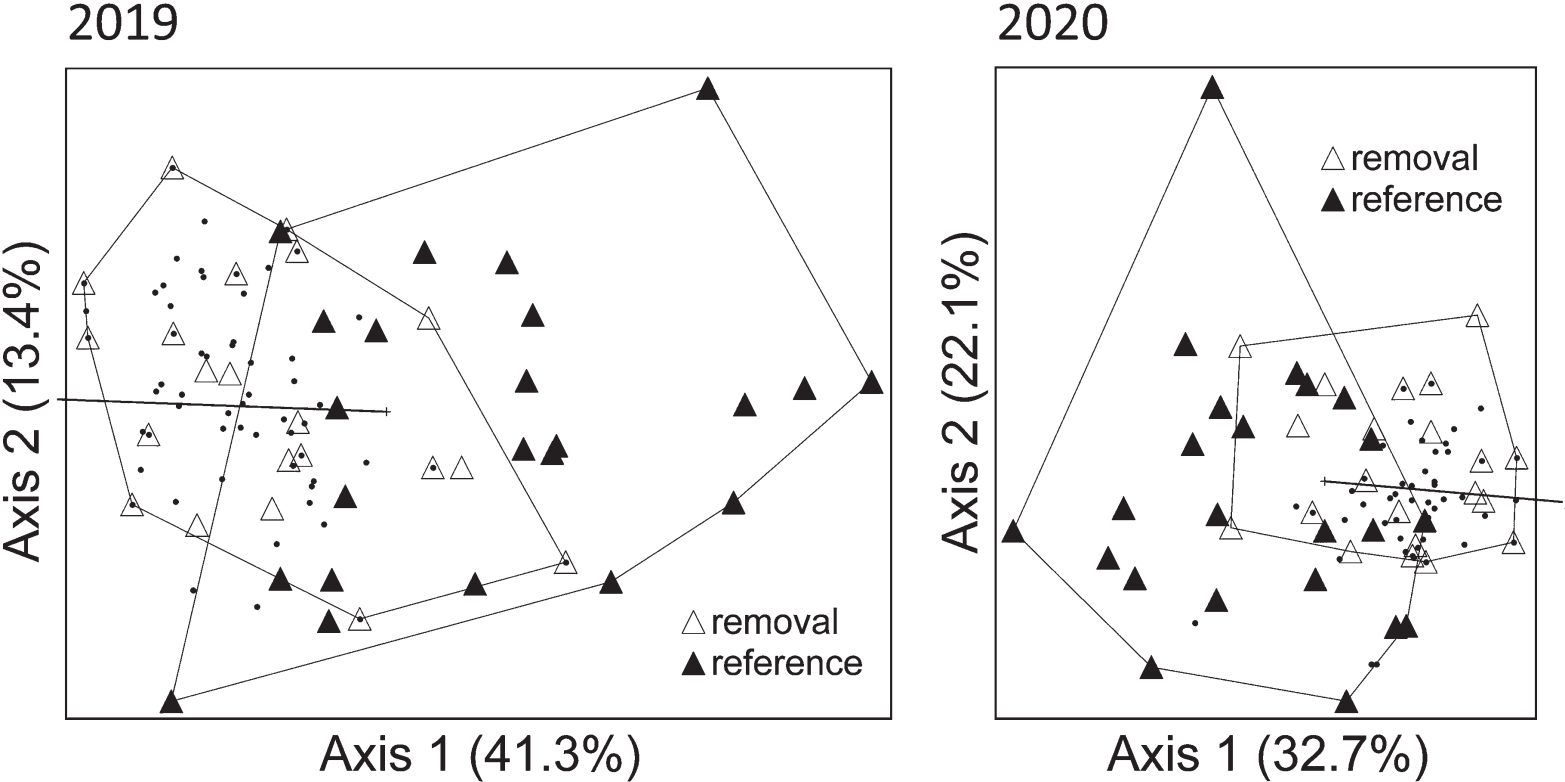
Ordinations from nonmetric multidimensional scaling showing differences in bee community composition among the sampling plots for 2019 and 2020 (note: a useful ordination was not found for 2018). Open and closed triangles represent removal and reference plots, respectively, while dots indicate individual bee species. The vector for bee richness is included in each ordination

Our NMDS analyses failed to produce a useful ordination for bees in 2018 but yielded three‐dimensional solutions for 2019 and 2020 (final stress = 16.74 and 16.97, respectively). Only the two axes explaining the most variation are presented in Figure 4, which for both years shows considerable overlap in bee communities between the two watersheds. According to MRPP, bee communities differed significantly between watersheds in the years following the prescribed fire, 2019 (*T* = −7.40, *p* < .001) and 2020 (*T* = −7.04, *p* < .001), but did not differ from one another after cutting, 2018 (*T* = −0.82, *p* = .19). Indicator species analysis identified 19 bee species that were significantly associated with one of the two watersheds (Table 3). Eighteen were associated with the removal watershed beginning in 2019. Only one species, *Bombus impatiens*, was associated with the reference watershed and this was only the case in 2018.

**TABLE 3:** Results from indicator species analysis (see methods) showing significant indicator bee (above) and plant (below) taxa for each year and treatment

	Removal			Reference		
	2018	2019	2020	2018	2019	2020
**Bees**						
*Andrena carolina* Viereck			50.9***			
*Andrena imitatrix* Cresson		19*	30.8*			
*Andrena pruni* Robertson			31*			
*Andrena uvulariae* Mitchell			19*			
*Augochlora pura* (Say)		61.7***	78.5***			
*Bombus impatiens* Cresson				36.4**		
*Lasioglossum abanci* (Crawford)		68.2***	59.5*			
*Lasioglossum cattellae* (Ellis)		31.5*	47.6***			
*Lasioglossum cressonii* (Robertson)		55.7*				
*Lasioglossum laevissimum* (Smith)		64.9***				
*Lasioglossum oblongum* (Lovell)		55.9***				
*Lasioglossum quebecense* (Crawford)		70**				
*Lasioglossum subviridatum* (Cockerell)		71.6***				
*Nomada luteoloides* Robertson		33.4*	25.9*			
*Nomada* sp. 10		33.1*	32.2*			
*Nomada* sp. 14		28.6**				
*Nomada* sp. 4		34*				
*Nomada* sp. 8		33.3**				
*Sphecodes coronus* Mitchell			19*			
**Plants**						
*Betula lenta* L.			19*			
*Liriodendron tulipifera* L.		31.6*				
*Pyrularia pubera* Michx.	25*	26.3*	24.8*			
*Rhododendron maximum* L.				36.4**	38.9**	30.4*
*Smilax rotundifolia* L.			39.3*			
Unknown cotyledon		31.6*				

Note

Indicator values are given with asterisks denoting the significance level: *p < .05; **p < .01, ***p < .001.

### 3.2 Plants

**FIGURE 5:**
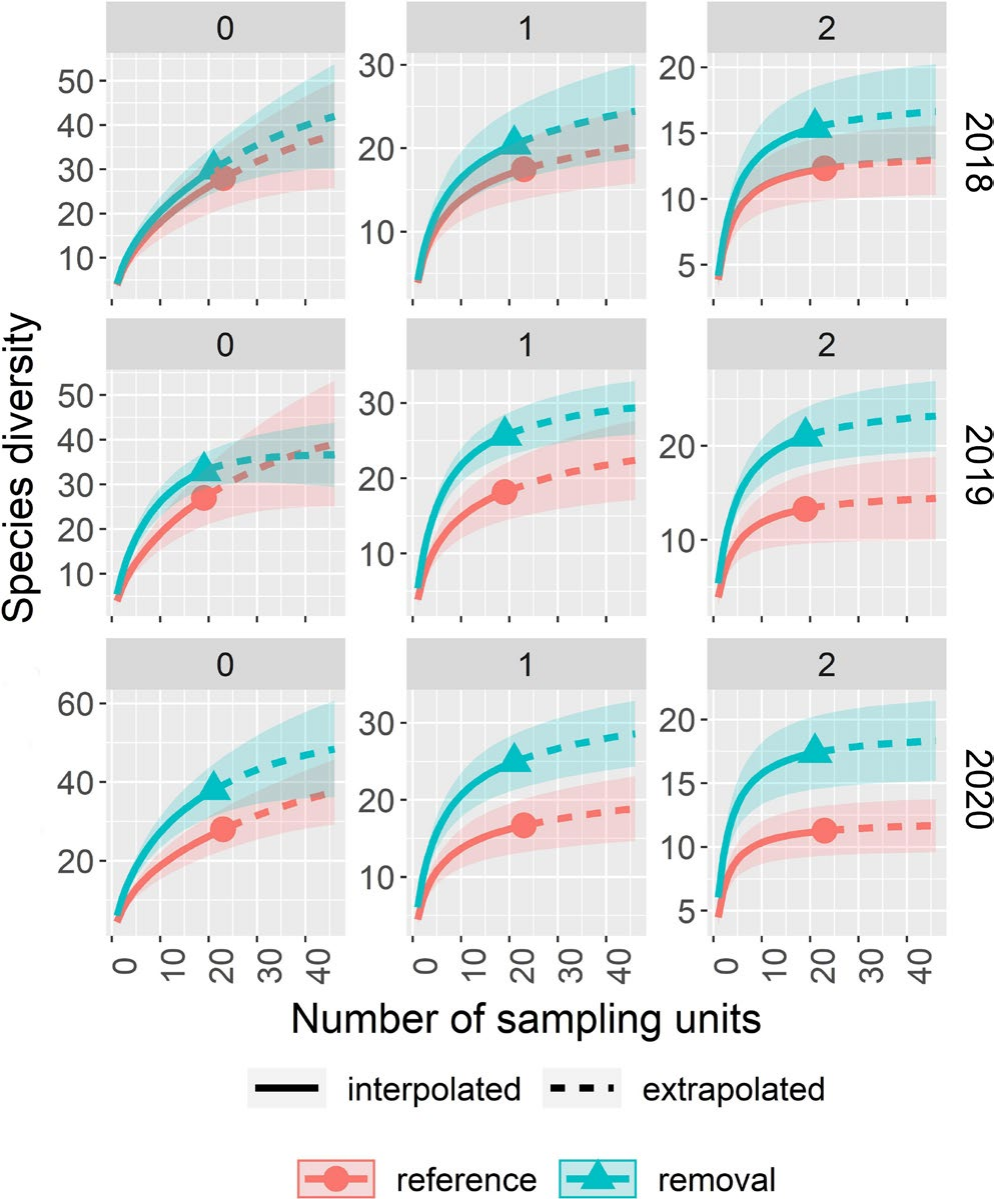
Rarefaction (solid lines) and extrapolation (dashed lines) of plant diversity in the reference and removal watersheds. Separate results are given for Hill numbers 0, 1, and 2. The results for species richness (*q* = 0) are shown in the left‐most panels, whereas those for Shannon's diversity (*q* = 1) and Simpson's diversity (*q* = 2) are shown to the right. All curves include 95% confidence intervals, and comparisons are made at twice the smallest reference sample size (i.e., 42 sampling units for 2018 and 2020 and 38 units for 2019)

A total of 54 plant species were recorded in our herb plots over the course of the study (Appendix 6). Similar to the results for bees, we found no differences in plant richness (*q* = 0), Shannon's diversity (*q* = 1), or Simpson's diversity (*q* = 2) between watersheds in 2018 based on overlapping confidence intervals at the base sample size (Figure 5). We only found significant differences between watersheds for *q* = 2 in 2019. In 2020, we still found no significant difference in plant richness between watersheds, but Shannon's diversity (*q* = 1) and Simpson's diversity (*q* = 2) were both significantly higher in the removal watershed (Figure 5). Our separate comparison of *R*. *maximum* cover categories provides more insight into how plant diversity is affected by the presence *R*. *maximum*. There were no differences among the three categories in 2018 although it is noteworthy that the absent plots accumulated species more quickly than the removed plots (Appendix 7). In 2019, plant richness (*q* = 0) was significantly higher in absent plots than in present plots and Shannon's diversity (*q* = 1) and Simpson's diversity (*q* = 2) were both significantly higher in the absent and removed categories than in the present category. The results for 2020 were the same as those for 2019 except that Simpson's diversity only differed significantly between the absent and present plots (Appendix 7). As stated above, as plant richness increased, bee richness increased; but, plant richness was unaffected by canopy openness. Regardless of year, plant richness was unaffected by burn ratio (RdNBR, data not shown).

According to MRPP, plant communities differed significantly between watersheds in 2018 (*T* = −2.09, *p* = .03), 2019 (*T* = −3.53, *p* < .01), and 2020 (*T* = −2.54, *p* = .02). Indicator species analysis identified 6 plant species that were significantly associated with one of the two watersheds (Table 3). Only one species, *R*. *maximum*, was associated with the reference watershed.

### 3.3 Fire severity

The average (±SE) RdNBR for the removal (burned) watershed was 1032.97 ± 160.73 (range: 21.63 to 2565.60). Only one of our plots within this watershed was unburnt (RdNBR < 70). The average (±SE) RdNBR for the reference watershed was −95.42 ± 20.09 (range: −448.37 to 15.94) and was −133.50 ± 18.93 for the four open roadside plots.

## 4 DISCUSSION

This study aimed to explore the effects of *R*. *maximum* removal followed by prescribed fire on bee and herb‐layer plant communities. Our results suggest that both taxa generally benefited from the treatment, although these effects did not become apparent until 2019 after the prescribed fire. While this delayed response may have been observed even without the prescribed burn, the conclusion that applying fire following shrub removal more strongly benefits bees than shrub removal alone is consistent with the findings of a previous study. Campbell et al. (2007) found flower‐visiting insects to be significantly more abundant in plots that were both mechanically cleared of *Kalmia* and *R*. *maximum* in the shrub layer and subsequently burned than in plots that received just one or neither of these treatments. Many studies have reported significant increases in bee abundance and richness the year following a fire (Carbone et al., 2018; Ulyshen et al., 2021). Although this is often attributed to increased floral resource availability in recently burned areas, it could also simply be due to enhanced trap visibility against a charred background. The second explanation is more likely in this case considering our sampling took place soon after the burn in 2019, presumably too soon for differences in floral resource availability to come into play. Moreover, weaker differences in bee diversity between watersheds were observed in 2020, including no significant difference in species richness, after the plant community had had more time to recover from the burn.

When analyzing data from the removal watershed separately, we found significant positive correlations between bee richness and fire severity (RdNBR) (Table 2). Although this is consistent with previous studies (Galbraith et al., 2019), the fact that this was true even in 2018, the year before the burn, suggests that some correlate of fire severity, rather than fire severity itself, is likely responsible for these relationships. Because plant richness, canopy openness, and elevation were not significantly correlated with RdNBR, these metrics can be ruled out as factors driving these patterns. Although not measured in this study, it is possible that downed woody debris may have resulted in both higher local fire severity as well as higher bee richness. Dead wood represents one of the most important resources to forest insect communities (Ulyshen, 2018) and is known to provide critical nesting sites for several of the bee species captured in this study (e.g., *Lasioglossum subviridatum* (Cockerell), *L*. *coeruleum* (Robertson), *Augochlora pura* (Say), and *Osmia* spp.). This possibility is further supported by previous studies documenting higher bee diversity in areas with abundant dead wood (Grundel et al., 2010; Loy et al., 2020).

The responses of plants to *R*. *maximum* removal and prescribed fire were similar to, but less dramatic than, those for bees. Although plant diversity did not differ between watersheds at any level of *q* in 2018 with only *R*. *maximum* removal, the diversity of common species was significantly higher in the removal watershed in 2019 and 2020 after the addition of prescribed fire. It is well established that fire can have a rejuvenating effect on herbaceous vegetation, resulting in increased diversity (Elliott & Miniat, 2018; He et al., 2019). Our results are consistent with this general pattern, but the strongest responses were seen among the more common species (Figure 5). Our comparisons of plant diversity among the different *R*. *maximum* cover classes show clearly that herbaceous plant diversity is higher in areas without *R*. *maximum*, especially soon after a fire. Interestingly, by 2020 the rarefaction and extrapolation curves for plots from which *R*. *maximum* was absent or removed were nearly identical, suggesting a rapid recovery of herbaceous plant species richness following the removal of the shrub.

We found bee richness and plant richness to be significantly correlated in all three years. This is more likely due to both groups being more species rich in relatively open areas free from *R*. *maximum* than to bees responding directly to differences in plant richness. Many of the plant species recorded in our plots were either wind‐pollinated species, too young to flower or were not observed flowering during the course of this study. Based on our general observations, floral resource availability is much lower within the closed forest than in clearings where we commonly observed bees actively visiting flowers. Although our sampling intensity was eleven‐fold greater in the two watersheds combined than in the roadside clearings in 2019, the clearings yielded almost as many species (66 vs 67) and over a third as many individuals. These findings suggest that roadsides and other clearings may be particularly important foraging habitats for bees in the southern Appalachians and perhaps other regions dominated by mature closed‐canopy forests.

We captured a diverse assemblage of bees visiting *R*. *maximum* flowers in this study, consisting of 27 species, and estimate that a quarter of all bee species in our study area utilize this floral resource. Among these species is at least one species, *Andrena cornelli* Viereck, that specializes on *R*. *maximum* and closely related taxa. Although we did not sample bees visiting *R*. *maximum* blooms within the forest, it is clear from these results that *R*. *maximum* provides an important resource to bee communities in the southern Appalachians.

## 5 CONCLUSIONS

*Rhododendron maximum* has benefited from the past century of disturbance in the southern Appalachians and is a more dominant member of the shrub layer than it was historically. The findings from this study suggest that both bees and herb‐layer plant diversity will increase to some degree from local efforts to reduce *R*. *maximum* cover and reintroduce fire. The benefits of such measures to bees appear to be relatively mild and short term, however, especially compared to the dramatic increases in plant and bee diversity reported after the eradication of *Ligustrum sinense* Lohr, a non‐native shrub (Ulyshen et al., 2022). Most notably, we only detected a significant increase in bee species richness (*q* = 0) in 2019, the season immediately following the fire, whereas no significant differences in richness were observed one year before or one year later. The fact that approximately one quarter of bee species in this study were estimated to visit *R*. *maximum* flowers suggests the removal of this shrub may even have some negative effects on bees and perhaps other pollinators. However, it should be noted that these data came from flowers growing in open roadside areas and may not accurately reflect the value of *R*. *maximum* flowers to bees in the shady environment of closed‐canopy forests. Taken together, our results suggest that removing *R*. *maximum* from forests has a detectable but perhaps short‐term benefit to bees if combined with prescribed fire. While our data may not specifically support the widespread removal of *R*. *maximum* for the purpose of improving pollinator habitat, this may be a helpful step for managers aiming to apply prescribed fire. Prescribed fire, in turn, has the potential to benefit bees over longer time periods by thinning the overstory and increasing the availability of floral resources near the forest floor. Our results further suggest that creating openings with wildflowers may greatly benefit bees in heavily forested areas of the southern Appalachians.

## CONFLICT OF INTEREST

The authors declare there are no competing interests.

## AUTHOR CONTRIBUTIONS

**Michael Ulyshen:** Conceptualization (equal); Data curation (lead); Formal analysis (lead); Investigation (lead); Methodology (equal); Writing – original draft (lead). **Katherine Elliott:** Conceptualization (equal); Investigation (supporting); Methodology (lead); Supervision (equal); Writing – review & editing (supporting). **Joel Scott:** Investigation (supporting); Writing – review & editing (supporting). **Scott Horn:** Investigation (supporting); Writing – review & editing (supporting). **Patsy Clinton:** Investigation (supporting); Writing – review & editing (supporting). **Ning Liu:** Formal analysis (supporting); Writing – review & editing (supporting). **Chelcy Miniat:** Conceptualization (equal); Project administration (equal); Writing – review & editing (supporting). **Peter Caldwell:** Conceptualization (equal); Project administration (equal). **Chris Oishi:** Conceptualization (equal); Project administration (equal). **Jennifer Knoepp:** Conceptualization (equal); Project administration (equal). **Paul Bolstad:** Conceptualization (equal); Project administration (equal).

## Data Availability

Data have been archived in Dryad: https://doi.org/10.5061/dryad.4xgxd25bv

## APPENDIX 1

Colored pan traps filled with soapy water were used to sample bees in the forest (left) and in roadside clearings (right)

## APPENDIX 2

*Rhododendron maximum* flowers can be found within forests, as shown here, as well as in more open areas

## APPENDIX 3

List of bee species collected in the reference and removal watersheds (with abundances for years 2018/2019/2020) as well as in open areas (2019 only). Asterisks indicate species also captured from *R*. *maximum* flowers growing near the watersheds (see Appendix 4)

**Table:**

Species	Reference	Removal	Open	Total
*Andrena carlini* Cockerell	0/3/2	0/1/0	2	8
*Andrena carolina* Viereck	1/0/1	0/3/31	0	36
*Andrena ceanothi*? Viereck	0/0/0	0/0/1	0	1
*Andrena cornelli* Viereck*	1/0/0	2/3/1	1	8
*Andrena crataegi* Robertson	0/0/0	0/0/0	2	2
*Andrena fenningeri* Viereck	0/0/0	0/0/0	1	1
*Andrena forbesii* Robertson	0/0/0	0/0/0	1	1
*Andrena heraclei* Robertson	0/0/2	0/2/7	1	12
*Andrena illini*? Bouseman and LaBerge	0/0/0	0/0/0	6	6
*Andrena imitatrix* Cresson	0/0/1	0/5/11	19	36
*Andrena mandibularis* Robertson	0/1/0	0/1/2	2	6
*Andrena melanochroa* Cockerell	0/0/0	0/1/0	0	1
*Andrena milwaukeensis* Graenicher	3/0/3	0/1/0	0	7
*Andrena miserabilis* Cresson	0/1/2	0/1/0	3	7
*Andrena nivalis* Smith	0/1/2	0/2/2	7	14
*Andrena nuda* Robertson	0/0/1	0/0/0	0	1
*Andrena perplexa* Smith	2/0/0	4/0/0	5	11
*Andrena pruni* Robertson	0/1/1	1/6/12	2	23
*Andrena robertsonii* Dalla Torre	0/0/0	0/0/1	0	1
*Andrena rugosa* Robertson	0/0/2	0/0/1	0	3
*Andrena sayi* Robertson	0/0/0	0/2/1	5	8
*Andrena spiraeana* Robertson	2/5/4	2/2/3	0	18
*Andrena uvulariae* Mitchell	0/1/0	0/0/4	0	5
*Andrena vicina* Smith	0/2/0	1/1/0	5	9
*Andrena violae* Robertson	1/3/1	0/1/0	3	9
*Andrena virginiana* Mitchell	1/0/0	0/0/1	0	2
*Andrena ziziaeformis* Cockerell	0/5/1	0/1/0	5	12
*Apis mellifera* L.*	0/0/0	1/1/4	0	6
*Augochlora pura* (Say)*	1/5/68	3/29/227	21	354
*Augochlorella aurata* (Smith)	0/0/1	0/1/3	14	19
*Augochloropsis metallica* (Fabricius)*	3/0/3	3/1/8	7	25
*Bombus bimaculatus* Cresson*	0/4/7	0/6/6	3	26
*Bombus citrinus* (Smith)	0/0/0	0/1/0	0	1
*Bombus griseocollis* (De Geer)	0/0/0	0/0/0	2	2
*Bombus impatiens* Cresson*	11/3/11	0/1/11	3	40
*Bombus perplexus* Cresson*	0/1/0	0/0/0	0	1
*Bombus sandersoni*/*vagans**	7/17/21	1/22/34	6	108
*Ceratina calcarata* Robertson	0/0/1	3/0/0	0	4
*Ceratina strenua* Smith	0/0/0	0/1/0	0	1
*Coelioxys rufitarsis* Smith*	0/1/0	0/0/2	0	3
*Eucera rosae* (Robertson)	0/0/0	0/0/1	0	1
*Habropoda laboriosa* (Fabricius)	0/0/0	0/0/0	1	1
*Halictus confusus* Smith	0/0/0	0/0/1	0	1
*Halictus rubicundus* (Christ)	0/0/0	0/0/1	0	1
*Hylaeus sparsus* (Cresson)	0/0/0	2/1/0	0	3
*Lasioglossum (D*.*) abanci* (Crawford)*	175/115/99	46/225/133	34	827
*Lasioglossum (H*.*) birkmanni* (Crawford)	0/0/0	0/0/1	0	1
*Lasioglossum (D*.*) callidum* (Sandhouse)	0/0/0	0/1/0	0	1
*Lasioglossum (D*.*) cattellae* (Ellis)	0/1/0	4/16/20	1	42
*Lasioglossum (D*.*) coeruleum* (Robertson)*	11/2/2	14/8/4	2	43
*Lasioglossum (L*.*) coriaceum* (Smith)	5/7/7	0/3/0	1	23
*Lasioglossum (D*.*) cressonii* (Robertson)*	14/33/46	10/56/32	5	196
*Lasioglossum foxii* (Robertson)	0/0/0	0/1/0	0	1
*Lasioglossum (D*.*) hitchensi* Gibbs*	0/0/0	0/0/0	1	1
*Lasioglossum (D*.*) laevissimum* (Smith)	3/9/4	3/47/6	1	73
*Lasioglossum (D*.*) nigroviride* (Graenicher)*	0/0/0	1/0/0	0	1
*Lasioglossum (D*.*) oblongum* (Lovell)*	3/1/2	16/42/18	14	96
*Lasioglossum (S*.*) quebecense* (Crawford)	25/97/139	23/245/149	191	869
*Lasioglossum (D*.*) smilacinae* (Robertson)*	0/0/0	1/0/0	0	1
*Lasioglossum (D*.*) subviridatum* (Cockerell)*	14/64/57	8/147/70	12	372
*Lasioglossum tegulare/puteulanum*	0/0/0	0/1/0	0	1
*Lasioglossum (D*.*) versans* (Lovell)	3/10/14	4/11/16	6	64
*Lasioglossum (D*.*) versatum* (Robertson)	0/0/3	0/0/1	2	6
*Lasioglossum* sp.	0/0/0	0/1/0	0	1
*Megachile gemula* Cresson*	1/0/0	1/0/2	2	6
*Megachile mendica* Cresson	0/0/0	0/1/0	2	3
*Nomada imbricata* Smith	2/0/0	0/0/0	1	3
*Nomada luteoloides* Robertson	0/2/1	0/13/9	4	29
*Nomada sulphurata* Smith	0/0/0	0/1/0	0	1
*Nomada* sp1	0/0/0	0/0/0	2	2
*Nomada* sp2	0/0/0	0/0/1	0	1
*Nomada* sp3	0/0/0	0/0/0	3	3
*Nomada* sp4	0/6/6	2/21/14	30	79
*Nomada* sp5	0/0/0	1/0/0	0	1
*Nomada* sp6	0/0/0	0/1/0	0	1
*Nomada* sp7	1/0/1	0/2/0	1	5
*Nomada* sp8	0/0/0	0/12/1	9	22
*Nomada* sp9*	4/3/2	0/5/3	10	27
*Nomada* sp10	0/2/2	2/12/10	3	31
*Nomada* sp11*	0/6/2	2/3/3	4	20
*Nomada* sp12	0/0/0	0/0/0	1	1
*Nomada* sp13	0/0/0	0/2/1	4	7
*Nomada* sp14	0/0/3	0/7/2	2	14
*Nomada* sp15	0/0/0	1/0/0	1	2
*Nomada* sp16	1/0/0	0/0/0	0	1
*Osmia albiventris* Cresson	0/0/0	0/1/0	2	3
*Osmia atriventris* Cresson	0/0/1	0/0/0	0	1
*Osmia bucephala* Cresson	0/1/2	0/2/0	1	6
*Osmia chalybea* Smith	0/0/0	0/1/0	3	4
*Osmia collinsiae* Robertson	0/0/0	0/0/0	1	1
*Osmia cornifrons* (Radoszkowski)	0/2/0	0/5/2	7	16
*Osmia distincta* Cresson	0/0/0	0/1/0	0	1
*Osmia georgica* Cresson	0/0/0	0/2/0	14	16
*Osmia pumila* Cresson	0/0/0	0/2/2	2	6
*Osmia sandhouseae* Mitchell	0/0/0	0/1/0	1	2
*Osmia taurus* Smith	1/1/1	0/1/0	1	5
*Pseudopanurgus virginicus* (Cockerell)	0/0/0	0/0/0	1	1
*Sphecodes coronus* Mitchell	0/1/0	0/2/4	1	8
*Sphecodes heraclei* Robertson	0/0/0	0/0/0	1	1
*Sphecodes levis* Lovell and Cockerell	0/0/1	0/4/5	0	10
*Sphecodes ranunculi* Robertson	0/0/1	0/0/0	0	1
*Sphecodes townesi* Mitchell	0/0/0	1/2/0	0	3
*Xylocopa virginica* (Linnaeus)	0/0/0	0/0/0	1	1
Total number of individuals	296/417/531	163/1005/885	512	3809
Total number of species	26/34/42	28/64/50	66	103

## APPENDIX 4

List of bee species collected from *R*. *maximum* flowers with total abundance for all years combined (2018–2020). Asterisks indicate species also captured in pan traps (see Appendix 3)

**Table:**

Species	Number
*Andrena cornelli* Viereck*	24
*Anthophora abrupta* Say	4
*Apis mellifera* L.*	10
*Augochlora pura* (Say)*	23
*Augochloropsis metallica* (Fabricius)*	1
*Bombus bimaculatus* Cresson*	4
*Bombus impatiens* Cresson*	2
*Bombus perplexus* Cresson*	2
*Bombus sandersoni*/*vagans**	16
*Coelioxys rufitarsis* Smith*	1
*Lasioglossum (D*.*) abanci* (Crawford)*	1
*Lasioglossum (D*.*) coeruleum* (Robertson)*	10
*Lasioglossum (D*.*) cressonii* (Robertson)*	1
*Lasioglossum fattigi* (Mitchell)	6
*Lasioglossum (D*.*) hitchensi* Gibbs*	1
*Lasioglossum imitatum* (Smith)	2
*Lasioglossum (D*.*) nigroviride* (Graenicher)*	1
*Lasioglossum (D*.*) oblongum* (Lovell)*	18
*Lasioglossum pectorale* (Smith)	2
*Lasioglossum pilosum* (Smith)	2
*Lasioglossum (D*.*) smilacinae* (Robertson)*	1
*Lasioglossum (D*.*) subviridatum* (Cockerell)*	8
*Lasioglossum viridatum* (Lovell)	1
*Lasioglossum zephyrum* (Smith)	1
*Megachile gemula* Cresson*	1
*Nomada* sp. 11*	2
*Nomada* sp. 9*	1

## APPENDIX 5

Rarefaction (solid lines) and extrapolation (dashed lines) of bee diversity in plots from which *R*. *maximum* was absent, present, or removed. Separate results are given for Hill numbers 0, 1, and 2. The results for species richness (*q* = 0) are shown in the left‐most panels, whereas those for Shannon's diversity (*q* = 1) and Simpson's diversity (*q* = 2) are shown to the right. All curves include 95% confidence intervals, and comparisons are made at twice the smallest reference sample size (i.e., 20 sampling units)

## APPENDIX 6

List of plant species recorded within the herb plots (within 0.5 m of the ground) in the reference and removal watersheds. The presence of a species is denoted with an x for each year

**Table:**

Species	Floral resource in herb layer?	Reference			Removal		
		2018	2019	2020	2018	2019	2020
*Acer pensylvanicum* L.	No		x		x	x	x
*Acer rubrum* L.	No	x	x	x	x	x	x
*Adiantum pedatum* L.	No				x	x	x
*Amelanchier arborea* (F. Michx.) Fernald	No	x	x	x	x	x	x
*Betula lenta* L.	No					x	x
*Betula* spp.	No	x		x		x	
*Calycanthus floridus* L.	No				x		x
*Carya glabra* (Mill.)	No				x	x	x
*Castanea dentata* (Marsh.) Borkh.	No				x	x	x
*Chimaphila maculata* (L.) Pursh	Yes	x	x	x	x		x
*Conopholis americana* (L.) Wallr.	No		x				
*Dichanthelium* spp.	Yes					x	
*Epigaea repens* L.	Yes	x	x	x			
*Erechtites hieraciifolius* (L.) Raf. ex DC	No						x
*Galax urceolata* (Poir.) Brummitt	Yes	x	x	x	x		x
*Gaylussacia ursina* (M. A. Curtis) Torr. & A. Gray	No	x	x	x	x	x	x
*Goodyera pubescens* (Willd.) R. Br.	Yes	x	x	x	x	x	x
*Hamamelis virginiana* L.	No	x	x	x			
*Kalmia latifolia* L.	No	x	x	x	x		x
*Liriodendron tulipifera* L.	No					x	x
*Magnolia acuminata* (L.) L.	No				x		
*Medeola virginiana* L.	Yes				x	x	
*Melampyrum lineare* Desr.	Yes		x	x	x		x
*Mitchella repens* L.	Yes		x				
*Neottia cordata* (L.) Rich.	No						x
*Nyssa sylvatica* Marshall	No	x	x	x		x	x
*Orchis spectabilis* (Schlegel)	Yes					x	x
*Oxydendrum arboretum* (L.) DC.	No	x	x	x			
*Polystichum acrostichoides* (Michx.) Schott	No				x	x	x
*Prenanthes* spp.	No	x	x	x	x	x	x
*Prunus serotina* Ehrh.	No		x	x			
*Pseudognaphalium obtusifolium* (L.) Hilliard & B. L. Burtt	Yes				x	x	x
*Pyrularia pubera* Michx.	No			x	x	x	x
*Quercus coccinea* (Dyar)	No	x	x	x	x		x
*Quercus montana* Willd.	No	x	x	x	x	x	x
*Quercus rubra* L.	No	x	x	x	x	x	x
*Quercus velutina* Lam.	No	x					
*Rhododendron maximum* L.	No	x	x	x			
*Robinia pseudoacacia* L.	No	x				x	
*Sassafras albidum* (Nutt.) Nees	No	x				x	x
*Smilax glauca* Walter	No	x	x	x	x	x	x
*Smilax rotundifolia* L.	No	x	x	x	x	x	x
*Solidago* sp.	Yes	x	x	x			
*Symplocos tinctoria* (L.) L’Hér.	No	x	x	x	x	x	x
*Thelypteris noveboracensis* (L.) Nieuwl.	No	x	x	x	x	x	x
*Uvularia puberula* Michx.	Yes						x
*Vaccinium* spp.	Yes	x	x	x			
*Veratrum parviflorum* Michx.	Yes				x	x	x
*Viola canadensis* L.	Yes				x		x
*Viola hastata* Michx.	Yes	x		x		x	x
*Viola rotundifolia* Michx.	Yes				x	x	x
*Viola* spp.	Yes				x	x	
*Vitis* spp.	No					x	x
Unknown cotyledon	No	x		x		x	x
Number of species		27	27	27	30	32	37

## APPENDIX 7

Rarefaction (solid lines) and extrapolation (dashed lines) of plant diversity in plots from which *R*. *maximum* was absent, present, or removed. Separate results are given for Hill numbers 0, 1, and 2. The results for species richness (*q* = 0) are shown in the left‐most panels, whereas those for Shannon's diversity (*q* = 1) and Simpson's diversity (*q* = 2) are shown to the right. All curves include 95% confidence intervals, and comparisons are made at twice the smallest reference sample size (i.e., 20 sampling units for 2018 and 2020 and 18 sampling units for 2019)
